# Supplementary material for: The Surplus Transplant Lung Allocation System in Italy: An Evaluation of the Allocation Process via Stochastic Modeling
Source: Int J Environ Res Public Health. 2021 Jul 3;18(13):7132. doi: 10.3390/ijerph18137132 (PMC8296876; doi:10.3390/ijerph18137132)
Supplement: Supplementary file 1 [file ijerph-18-07132-s001.zip › ijerph-1231117-supplementary.pdf]

Supplementary material

S1 Probability models

1. Probability model

There are two probability models. The first one uses conditional probabilities based on the protocol flowchart depicted in Figure 1 to compute the probability for each region to receive (i.e., to be asked and accept it) each n-th organ in the period considered. The second probability model uses all those probabilities to compute the probability of receiving at least k organs out of the N provided during the period considered. The latter takes advantage of the binomial distribution to correlated binary variables based on Bahadur's representation [2].

Definition 1.1

(Variable's convention). Let A be the set of macroareas in the state. For our purpose and for all the probability models actually considered, that set contains just two elements, e.g., "North" and "South".

For $\delta \epsilon A$, let n_δ be the number of regions with at least one transplant center in macroarea δ, and m_δ be the number of regions without any transplant centers in macroarea δ.

Let $C^\delta = \left\{ c^\delta_{i_\delta} : i_\delta \in \{1, \dots, n_\delta\} \right\}$ be the set of (macro)regions with at least one transplant center in macroarea δ.

Let $O^\delta = \left\{ o^\delta_{i_\delta} : i_\delta \in \{1, \dots, m_\delta\} \right\}$ be the set of regions without any transplant center in macroarea δ.

Let P^X be the probability that a surplus organ is provided by a region $x \in X$ where X is simply a generic set of regions.

Define similarly P^x as the probability that a surplus organ is provided by a (supposed and well defined) region x.

Let P_X be the probability that if a surplus organ is offered to the set of region X, it will be used by some $x \in X$ (without any consideration of the criteria leading the decision of which $x \in X$ uses it). Define

similarly P_x as the probability that a surplus organ offered to a (supposed and well defined) region x is accepted by x itself.

By protocol rules, a region that produces a surplus organ cannot be further asked to use it. That said, without any consideration of the macroregion strips but only considering that a surplus organ is purposed with a priority for the regions in the macroregions that produce it, we can compute the probability that a surplus organ will be effectively used, adding the probability for each macroarea to be selected to ask its regions to use the organ, and that at least one of them (excluding the one that possibly has produced it) accepts it.

Proposition 1.1 (Overall probability of using a surplus organ). With the notation in Definition 1.1, the probability for a surplus organ to be used is:

$$\sum_{\delta \in A} \left[P_{C^{\delta}} \left[P^{O^{\delta}} + P^{O^{1-\delta}} \left(1 - P_{C^{1-\delta}}\right) + \sum_{i \in n_{\delta}} P^{c^{1-\delta}_{i_{1-\delta}}} \left(1 - P_{C^{1-\delta} \setminus c^{1-\delta}_{i_{1-\delta}}}\right) \right] + \sum_{i \in n_{\delta}} \left(1 - P_{C^{\delta} \setminus c^{\delta}_{i_{\delta}}}\right) \right]$$

From the equation reference above, the total, by the computation of its complement to 1, it is straightforward to obtain the probability of losing a provided surplus organ. Similarly, taking each of the components of the main summation, it is possible to compute the probability that each macroarea has to use the organ.

2. Macroarea level

With the aim of computing the dynamics inside each macroarea, i.e., what are the probabilities for each (macro)region in a macroarea to receive (and so also accept) an offered surplus organ? It is necessary to know what is the probability that a (macro)region accepts if it has been offered. To this aim, it is also necessary to know what the probabilities for a (macro)region to be asked to use a surplus organ are, which directly depend on its current position in the strip and on the decisions made by the other (macro)regions possibly before it in the strip.

Definition 2.1

Let M the number of surplus organs supposed to exist in the period.

Let $t \in \{1, \dots, M\}$ be the t-th surplus organ provided in the period.

Let $T^{t,j_\delta}_{c^\delta_{i_\delta}}$ be the probability for the (macro)region $c^\delta_{i_\delta}$ to be at the position $j_\delta \in \{1, \dots, n_\delta\}$ of its strip while the organ t-th (out of M) is provided.

Let $P_{c^\delta_{i_\delta}, j_\delta}$ be the probability for the center $c^\delta_{i_\delta}$ to obtain (accepting it) a surplus organ if it is located at position j_δ in its strip.

Let S_x be the number of surplus organs provided by a region x.

With any effort, it is also possible to find the probability that a surplus organ is provided by a region not in a set of regions X by considering the ratio between the surplus organs provided by the other regions in the reference period and the overall amount of surplus organs offered in that period.

Remark (Probability that a surplus organ is provided from outside). For every set of region X, the probability that a surplus organ provided is provided by some region, not in X, is:

$$\overline{S_x} = 1 - \frac{S_x}{M}. \qquad (1)$$

In the case in which $X = \{x\}$ is a set counting only one region, we will write simply $\overline{S_x}$.

Remark. For every $\delta \epsilon A$ and every $i_\delta \in \{1, \dots, n_\delta\}$, the following equality holds:

$$P_{c^\delta_{i_\delta},1} = P_{c^\delta_{i_\delta}}$$

Lemma 2.1

(Probability of obtain a surplus organ at a given position on the strip). For every $\delta \epsilon A$ and every $i_\delta, j_\delta \in \{1, \dots, n_\delta\}$ the probability for the center $c^\delta_{i_\delta}$ to obtain (accepting it) a surplus organ if it is located at the position j_δ in its strip is provided by its probability of accepting a surplus organ if it is asked for, through multiplication by the product of the probability that every (macro)region before in the strip does not accept

it[1].

$$P_{c^{\delta}_{i_\delta, j_\delta}} = P_{c^{\delta}_{i_\delta}} \prod_{0<j<j_\delta} \left(1 - P_{c^{\delta}_{(1,\dots,n_\delta)^{i_\delta - j_\delta}_{i_\delta - j}}} \right) \qquad (2)$$

Following the continuous-strip model used in the Italian surplus management protocol, it is possible by induction on t to define $T^{t,j_\delta}_{c^{\delta}_{i_\delta}}$ for every macroarea $\delta \epsilon A$, every positions $i_\delta, j_\delta \in \{1, \dots, n_\delta\}$ and every (macro)region $c^{\delta}_{i_\delta} \in C^{\delta}$.

Lemma 2.2

(Probability of being at a given position at a given time). With the notation in Definition 1.1, the probability for a (macro)region to be at a given position into its strip at the time in which the given surplus organ is provided is inductively defined as follows for every $\delta \epsilon A$, $i_\delta, j_\delta \in \{1, \dots, n_\delta\}$ and every $c^{\delta}_{i_\delta} \in C^{\delta}$:

$$T^{1,j_\delta}_{c^{\delta}_{i_\delta}} = \begin{cases} 1 & if\ i_\delta = j_\delta \\ 0 & otherwise \end{cases} \qquad (3a)$$

$$T^{t+1,j_\delta}_{c^{\delta}_{i_\delta}} = \sum_{j=1}^{n_\delta} \left[T^{t,j}_{c^{\delta}_{i_\delta}} \cdot \prod_{\substack{k \neq 0 \\ k < n_\delta - j_\delta + j}} \left[\left(1 - P_{c^{\delta}_{(1,\dots,n_\delta)^{i_\delta - j_\delta}_{k}}} \right) P_{c^{\delta}_{(1,\dots,n_\delta)^{i_\delta - j_\delta}_{i_\delta - j_\delta}}} \right] \right] \qquad (3b)$$

Corollary 2.1

(Probability of obtaining a given surplus organ). For every surplus organ $t \in \{1, \dots, m\}$ provided, for every (macro)region $c^{\delta}_{i_\delta} \in C^{\delta}$ in a macroarea $\delta \epsilon A$, the probability for $c^{\delta}_{i_\delta}$ to obtain and accept the *t*-th surplus organ is:

---

[1] Note: in the equation 3b $(1, \dots, n)^{j}_{i}$ represent the *i*-th element of the permutation of degree *j* for the ordered set $(1, \dots, n)$.

$$P^t_{c^\delta_{i_\delta}} = \overline{S_{c^\delta_{i_\delta}}} \sum_{j=1}^{n_\delta} \left[T^{t,j_\delta}_{c^\delta_{i_\delta}} P_{c^\delta_{i_\delta}, j_\delta} \right] \qquad (4)$$

Now, taking advantage of the work done in [2], we can finally compute the probability of each (macro)region to obtain and accept at least k surplus organs for every $k \in \{1, \dots, M\}$.

Proposition 2.2.

(Probability for at least k surplus organs) For every $n \in N$, let $x^n = \{(x_{1,\dots,} x_n): x_i \epsilon \{0,1\}$ for every $i \in \{1, \dots, \mathrm{n}\}$ be the set of all possible sequences of n 0s and 1s. Let $\zeta(x^n) = \sum_{t=1}^{n} x_t$. Hence, for every (macro)region $c^\delta_{i_\delta}$in a macroarea $\delta \epsilon A$ and for every number $0 < k \leq M$, by Corollary 2.1 and the formulas in [2]; the probability for $c^\delta_{i_\delta}$ to obtain at least k surplus organs out of M in the period considered is:

$${}^M_k P_{c^\delta_{i_\delta}} = \sum_{x:\zeta(x^M)>k} \left[\prod_{t=1}^{M} \left[\left(P^t_{c^\delta_{i_\delta}} \right)^{x_t} \left(1 - P^t_{c^\delta_{i_\delta}} \right)^{1-x_t} \right] \right] \qquad (5)$$

From the equation reference above, it is possible to provide the probability of receiving and accepting at least k out of the M surplus organs for every (macro)region. It should be noted that at this level, a macroregion counts as a normal region, not as a macroregion, while the regions in the macroregions are never considered separately.

3. Macroregion level

The differences between the rules for the dynamics in the macroarea strips and the macroregion strips are that in the latter, the region can be considered more than once, the strip is updated every time, and some region in the macroregion accepts the organ purposed. Moreover, in the macroregion, the strip is updated only if the surplus organs are provided originally by a region, not in the macroregion itself. Hence, to look at the dynamics inside a macroregion, it must be considered that the update happens only if the

organ purposed comes from the outside of the microregion, and a region in the macroregion has accepted it and it does not matter which one. Moreover, the update, when it happens, is independent of which region caused it and is every time there is a switch of one position of all the strips. By these considerations, it is possible to use a simplified version of Equation 3b in which all the products inside the summation are substituted by the product of two fixed probabilities: (1) the probability that at least one region in the macroregion accepts a surplus organ offered to the macroregion and (2) the probability that that organ is provided by a region, not in the macroregion. Moreover, the whole summation can be eliminated considering that the probability of interest is the product of the probability of being in the very next position in the strip with respect to the position of interest, and the probability of an update of the strip to happen.

Before writing the formulas, it is useful to fix some notations.

Definition 3.1

(Variable's convention for macroregion). Let R be the set of macroregions in the nation.

For every $\tau \in R$ let n_τ be the number of regions with at least one transplant center in microregion τ, and m_τ be the number of regions without any transplant centers in microregion τ.[2] Moreover, let $\overline{n_\tau}$ be the length of the strip of τ.

Let $C^\tau = \left\{\mathrm{c}_{i_\tau}^\tau : i_\tau \in \{1, \dots, n_\tau\}\right\}$be the set of regions with at least one transplant center in microregion τ.

Let $I_{\mathrm{c}_{i_\tau}^\tau} \subset (1, \dots, , \overline{n_\tau})$ be the starting set of indexes in which $\mathrm{c}_{i_\tau}^\tau$ appears in the strip of τ.

Let $I_{\mathrm{c}_{i_\tau}^\tau}^t$ the set of indexes in which $\mathrm{c}_{i_\tau}^\tau$ appears when the *t*-th surplus organ is provided.

Let $O^\tau = \left\{\mathrm{o}_{i_\tau}^\tau : i_\tau \in \{1, \dots, m_\tau\}\right\}$ be the set of regions without any transplant centers in the macroregion τ.

For every $\tau \in$ R let $S_\tau = \sum_{C_\tau} S_{C_\tau} + \sum_{O_\tau} S_{O_\tau}$

[2] Clearly $n_\tau \leq \overline{n_\tau}$ but despite the case of the macroareas and state strip, the inequality can be strict in this case because inside the macroregions strips repetition are admitted.

It is straightforward, by considering the complementarity of the probability that every region in the macroregion does not accept a surplus organ offered to the macroregion, to compute the probability that at least one region of the microregion accepts a surplus organ offered to the macroregion.

Remark (Probability of a macroregion accepting an organ if offered). For every $\tau \in \mathrm{R}$ the probability that exists $c_\tau \in C^\tau$ that accepts a surplus organ offered to τ is:

$$P_\tau = 1 - \prod_{c_\tau \in C^\tau} \left(1 - P_{c_\tau}\right) \qquad (6)$$

With the elements provided by Equations 6 and 1, it is finally possible to provide the modified version of Equation 3b and to adapt it to compute the probability that at a given time, a given region in a given macroregion is located at a given position in its strip.

Lemma 3.1

(Probability of being at a given position at a given time into a macroregion). With the notation in Definitions 2.1 and 3.1, the probability for a region to be at a given position in the strip of its macroregion at the time in which the given surplus organ is provided, is inductively defined as follows for every $\tau \in$ R, $i_\tau \in \{1 \ldots, n_\tau\}$, $j_\tau \in \{1 \ldots, \overline{n_\tau}\}$ and every $\mathrm{c}_{i_\tau}^{\tau} \in \; C^\tau$:

$$T_{c_{i_\tau}^{\tau}}^{1,j_\tau} = \begin{cases} 1 & if\ i_\tau \in I_{c_{i_\tau}^{\tau}} \\ 0 & otherwise \end{cases} \qquad (7a)$$

$$T_{c_{i_\tau}^{\tau}}^{t+1,j_\tau} = T_{c_{i_\tau}^{\tau}}^{1,(1,\ldots,\overline{n_\tau})^2_{j_\tau}} P_\tau \overline{S}_\tau \qquad (7b)$$

Now, it is necessary to compute the probability for a region in a macroregion to accept a surplus organ offered to the macroregion if the region is at a given position in the strip of the macroregion. This computation is not straightforward because of the possible repetition of the regions in the macroregion strips. Regardless, it is sufficient to observe that only the "first", or "one", answer for a region matters; in fact, if it accepts the purposed surplus organ it has accepted it and no more regions (neither that one) will be asked to accept the same surplus organ. While, if it does not accept, it has no capability to consider a

further question to that region for the same surplus organ. This leads us to consider probability independence at the moment in which the strip is considered; in fact, a region can be at a certain same position at a different time but has a different set of other regions at the different times to consider. Moreover, it is necessary to consider that a region cannot accept a surplus organ provided by itself. Regarding the last consideration, it is sufficient to consider at all times the probability for a region to accept a surplus organ is to multiply it by the probability that that region did not provide that organ.

Lemma 3.2

(Probability of obtaining an organ at a given strip position in a macroregion). For every $\tau \in$ R and every $i_\tau \in \{1 \dots, n_\tau\}$, $j_\tau \in \{1 \dots, \overline{n_\tau}\}$, the probability for the center $c^\tau_{i_\tau}$ to obtain (accepting it) the t-th surplus organ if it is located at the position j_τ in its strip, is provided by its probability of accepting a surplus organ if it is asked, by multiplication by the probability that the organ is not produced in the region by the

product of the probability that every region possibly before it in the strip does not accept the organ:

$$P^t_{c^\tau_{i_\tau, j_\tau}} = P_{c^\tau_{i_\tau}} \overline{S_{c^\tau_{i_\tau}}} \cdot \prod_{c^\tau : I^t_{c^\tau} \cap \{0, \dots, j_\tau - 1\} \neq 0} (1 - P_{c^\tau}) \qquad (8)$$

Corollary 3.1

(Probability of obtaining a given surplus organ in a macroregion). For every surplus organ t $\in$ $\{1, \dots, M\}$ provided, for every region $c^\tau_{i\tau} \in C^\tau$ in a macroregion $\tau \in$ R, the probability for $c^\tau_{i_\tau}$ to obtain and accept the *t*-th surplus organ is:

$$P^t_{c^\tau_{i_\tau}} = \sum_{j_\tau = 1}^{n_\tau} \left[T^{t, j_\tau}_{c^\tau_{i_\tau}} P^t_{c^\tau_{i_\tau}, j_\tau} \right] \qquad (9)$$

The application of Equation 9 remains exactly the same.

Table S1 Number of lungs transplanted in 2015 and 2016 for each region.

		**Number of lungs transplanted**	
Macroregion	Region	2015	2016
MA NORD	NITp	43	38
	Piemonte	6	11
	Emilia Romagna	3	4
	Toscana	4	9
MA SUD	Lazio	5	7
	Sicilia	5	5
ITALIA		66	74

MA: Macroarea, NITp: North Italian Transplant program

Table S2 Lungs donor in 2015 and 2016

Region		2015				2016			
		**Proposed**	**Accepted**	**Transplanted**	**Accepting region**	**Produced**	**Accepted**	**Transplanted**	**Region of transplantation**
MA NORD	NITp	28	2	2	2 Piemonte	56	5	4	1 RM Umberto I-1 Siena- 1 Torino-1 Zurigo
	Piemonte	9	2	2	1 NITp, 1 Emilia	23	5	2	1 IsMeTT-1 Milano
	Emilia Romagna	15	6	5	3 NITp, 1 Piemonte, 1 Toscana	37	14	12	5 Torino – 4 MI Policlinico – 1 Siena- 2 Padova
	Toscana	6	1	1	1 NITp	29	9	4	1 Padova – 2 MI Policlinico- 1 Pavia
MA SUD	Sardegna	9	2	1	1 NITp	18	9	6	4 Torino-1 Padova-1 Pavia
	Abruzzo-Molise	8	4	3	2 NITp, 1 Piemonte	10	6	1	1 IsMeTT
	Basilicata	1	0	0		1	1	1	1 IsMeTT
	Calabria	5	1	0		12	4	2	1 Bergamo-1 Torino

Campania	24	5	3	1 Lazio, 1 Sicilia, 1 Piemonte	39	9	5	1 RM Umberto I-1 Milano-1 Padova-1 Pavia-1 Torino
Lazio	11	2	1	1 Piemonte	30	8	4	1 Bologna-2 Padova-1 IsMeTT
Puglia	15	7	4	4 Sicilia	16	5	3	1 Pavia – 1 MI Policlinico-1 IsMeTT
Sicilia	5	2	2	1 Emilia, 1 Spagna	8	0	0	-
Umbria	2	0	0		5	2	2	1 RM Umberto I – 1 MI Policlinico

MA: Macroarea, NITp: North Italian Transplant program, IsMeTT: Istituto Mediterraneo Trapianti
